# MRPack: Multi-Algorithm Execution Using Compute-Intensive Approach in MapReduce

**DOI:** 10.1371/journal.pone.0136259

**Published:** 2015-08-25

**Authors:** Muhammad Idris, Shujaat Hussain, Muhammad Hameed Siddiqi, Waseem Hassan, Hafiz Syed Muhammad Bilal, Sungyoung Lee

**Affiliations:** 1 Ubiquitous Computing Lab., Department of Computer Engineering, Kyung Hee University, Yongin-si, Gyeonggi-do, Republic of Korea; 2 Haptics Lab., Department of Computer Engineering, Kyung Hee University, Yongin-si, Gyeonggi-do, Republic of Korea; CNRS UMR7622 & University Paris 6 Pierre-et-Marie-Curie, FRANCE

## Abstract

Large quantities of data have been generated from multiple sources at exponential rates in the last few years. These data are generated at high velocity as real time and streaming data in variety of formats. These characteristics give rise to challenges in its modeling, computation, and processing. Hadoop MapReduce (MR) is a well known data-intensive distributed processing framework using the distributed file system (DFS) for Big Data. Current implementations of MR only support execution of a single algorithm in the entire Hadoop cluster. In this paper, we propose MapReducePack (*MRPack*), a variation of MR that supports execution of a set of related algorithms in a single MR job. We exploit the computational capability of a cluster by increasing the compute-intensiveness of MapReduce while maintaining its data-intensive approach. It uses the available computing resources by dynamically managing the task assignment and intermediate data. Intermediate data from multiple algorithms are managed using multi-key and skew mitigation strategies. The performance study of the proposed system shows that it is time, I/O, and memory efficient compared to the default MapReduce. The proposed approach reduces the execution time by 200% with an approximate 50% decrease in I/O cost. Complexity and qualitative results analysis shows significant performance improvement.

## Introduction

The exponential growth in the amount of data generated during the last few years have greatly changed ideas about the value, management, and expertise of such data [1]. As of 2012, about 2.5 exabyte data are created each day, and this doubles about every three years. The current amount of data generated each second is more than all of the collective data from the previous 20 years. In 2011, the human digital universe contained 1.7 Zbytes, and this dataset is expected to increase by almost five times by 2015 (7.9 Zbytes) [2]. Similarly, the speed of data generation is more important than its volume. Real-time or nearly real-time data streaming [3, 4] makes a system more agile. Big Data sources generate data in various formats such as images, audio, GPS signals, text, sensory data, and huge amount of social network information. Thus the conventional storage, processing, and modeling technologies such as RDBMS, and linear programming techniques are ill-suited for Big Data. Requirements for the hay stack of data processing and management in effective time and computational cost have made the data-intensive approach economical.

Data-intensive approaches tend to process the data “in-place” and broadcast the final or intermediate results. Many data-intensive approaches exist for Big Data; but Apache Hadoop is one of the best-known data-intensive frameworks. Hadoop is an open source implementation of Google’s MapReduce [5], which processes data in the distributed file system (HDFS). The MapReduce algorithm can write applications in a high level programming model and hide the details of a working program in a cloud (which has many clusters) of commodity hardware. It functions in two phases map and reduce. However, MapReduce has a limitation of running single algorithm on distributed data in parallel [6] as a single job for data intensive [7] applications. Due to this limitation, the whole cluster is engaged in processing a single algorithm as a batch process.

In compute-intensive applications, extensive computation on shared data and message passing between workers takes place in a highly coupled environment (e.g., image processing and weather information processing using high performance computing (HPC)). Optimization techniques in MapReduce try to maximize the use of computation resources and reduce I/O operations. In an effort to combine data-intensive solutions with compute-intensive solutions, we propose *MRPack*. Our motivation is to execute multiple algorithms on the same distributed data in a single MapReduce job rather than a single cluster. A motivational example would be predictive analysis where the same dataset will be used to train multiple statistical models such as the linear model (LM), generalized linear model (GLM), and auto-regressive integrated moving average (ARIMA); different computations will be performed on the same dataset or data split. This technique is time-, communication-, and memory-efficient.

The main contribution includes design and development of a variant of the MapReduce algorithm while extending the generic MapReduce approach to incorporate compute-intensiveness with improved performance and efficiency. Compute-intensiveness is ramped up in MapReduce by changing the implementation of mappers and reducers. In this approach, a single map and reduce worker executes mappers and reducers of all included algorithms in a job. To differentiate between intermediate data of various algorithms, a generic hierarchical and composite key structure is defined. A data skew mitigation strategy is adapted to avoid data skew and long-reducers processing. Prereducer sorting and combining is defined based on algorithmic keys and each algorithm output is generated as the output of a separate reducer.

The proposed approach improves performance using the following factors: decreasing map input (*Read*) by reading in a single main mapper function, and (*Write*) using a single main reducer function, custom data partitioning, and algorithmic key-based skew mitigation. The proposed solution shows (2*x*) performance improvement compared to Generic MapReduce in I/O communication.

## Related Work

Gartner espoused a view of Big Data as having three basic dimensions: volume, variety, and velocity. The International Data Corporation (IDC) holds that “Big Data technologies describe a new generation of technologies and architectures designed to economically extract value from very large volumes of a wide variety of data, by enabling high-velocity, capture, discovery, and/or analysis” [8]. Considering these and many other aspects such as veracity and complexity of data, many Big Data processing frameworks have been developed and adapted [9, 10].

Hadoop is a well-known open-source Big Data framework developed by Apache [5, 11] inspired from Google’s File System (GFS) and initially adapted by Yahoo [12]. This framework was designed to process large variety datasets (Big Data) and it used distributed file system (HDFS) as storage layer and MapReduce as its processing layer. Hadoop is well-known for its data-intensive approach of data processing using MapReduce.

In Big Data research, an operating environment consisting of homogeneous nodes, out-performs a heterogeneous cluster unless a robust technique is developed to deal with node heterogeneity. Amazon Elastic Cloud (EC2) [13] is an example of a heterogeneous cluster that consists of virtual machines with different configurations on possibly different physical machines. Many solutions have been presented to improve Hadoop performance in heterogeneous environment [14–17]. Mechanisms have been designed for task scheduling in MapReduce and parallel execution of map tasks and reduce tasks and sharing between individual mappers running on separate machines. In [15], the authors introduced the distributed meta data store (DMDS) to allow mappers of a job to share information. Chen et al. in [18], presented a task placement optimizer to dynamically identify failing tasks in a cluster. Similarly, in an effort to optimize MapReduce performance, previous studies [19, 20] and [21] have addressed the issues of local data execution on DataNodes in the cluster by executing map and reduce tasks on local data.

In [22], the authors proposed a high-level declarative model and its run-time. This model targets datasets that are inter-dependent and programs to monitor, compile, and execute on these dependent datasets. Valvag et al. [23] have presented Cogset, which proposes tight-coupling between distributed systems. It integrates a storage file system with its execution environment (i.e., tightly coupled). Oivos and Cogset do not target the same, homogeneous, and independent datasets. In an attempt to introduce work-sharing across jobs in MapReduce, MRShare has been proposed by Nykiel and Potamias et al. [21] to enable automatic and principled work-sharing by transforming a batch of queries into a new batch that can function as single merged query.

Hadoop MapReduce version 2.0 (YARN) provides resource management to the job life-cycle [24]. It separates the functions of scheduling and management, and provides sharing of a cluster among various applications used by organizations. In YARN, each algorithm is executed as a separate MapReduce job and cluster resources are managed accordingly. This framework does not support concurrent algorithm execution in a single MapReduce job and its performance compared to earlier versions of MapReduce is superior only in terms of jobs/applications management. YARN also supports job chaining, where an iterative algorithm can be executed as a chained job. The main limitation of YARN is that it does not support parallel execution of algorithms on the same data; rather, it needs to write a separate job for each algorithm.

Examining the literature reveals that most of the existing systems do not exploit vailable resources and add extra cost in terms of time and communication. Extra communication cost is incurred by input and output data movement for each algorithm and context switching between jobs.

In all the above discussed Big Data processing frameworks and techniques, MapReduce is the widely adopted framework. All of these approaches addressed MapReduce’s performance improvements; however, these approaches only cater a single algorithm as a MapReduce job incurring extra communication cost. These limitations lead us to propose *MRPack*, a MapReduce variant model to execute multiple algorithms in a single job. The goals of this work are to pack multiple algorithms in a single job rather than a single cluster. We present multiple algorithms execution in a single MapReduce job to incorporate compute-intensiveness which significantly improves the performance as discussed in the results section.

## Methodology

This section presents the main contribution of the paper: design and development of *MRPack*, a technique to bridge the compute-intensive and data-intensive approaches, improve the MapReduce performance, and provide an algorithm suite in MapReduce. In *MRPack*, we address limitations of the MapReduce framework and propose a MapReduce based technique to process data in parallel using multiple algorithms. The main idea and motivation of *MRPack* are to reduce the MapReduce execution time of several sequential algorithms by implementing them concurrently. In this model, users can write a single MapReduce job and execute multiple algorithms that process the same data. Each algorithm is implemented in Map and Reduce functions, thus extending the main Map and Reduce functions of the job. Data in HDFS are read only once by Mapper of the main job and written back to HDFS by the Reducer of the main job. Algorithms involved in job work as sub-jobs and intermediate data are differentiated using a key mechanism specified following the polymorphism and object composition technique of objected oriented programming (OOP). The following sections formalize all the concepts in detail. Part of the proposed work has been previously presented [25].

### Preliminaries

In this section, we discuss some preliminary terms and concepts that need to be understood before understanding the general concept of *MRPack*.

- Intermediate Data: The data generated by mappers in (key, val) pairs and communicated between DataNodes are known as intermediate data; such data i sgenerated by all algorithms involved in *MRPack*. Therefore, each data needs to be correctly organized.
- Data Skew: This occurs in intermediate data, when the data belonging to a specific key or algorithm with high frequency of high generation overthrow the average key distribution. This data skew delays or increases the completion time of a job, which is a waste of resources because the resources are waiting for the data.
- Composite Key: In *MRPack*, we propose a composite key structure to address the “one-key” limitation of MapReduce. MapReduce uses only one key, so multiple algorithms cannot be executed in it. In a hierarchical and composite key system, we design a generic base class for all keys and extend it for specific algorithm key requirement. The detailed description is presented in following sections.
- Sub-Map: In *MRPack*, a mapper of each algorithm is executed as a sub-part of the main Mapper. Consider, we have a set of algorithms *A* = *a*_1_,*a*_2_,*a*_3_,…,*a*_*n*_ to be executed, the Map function of each algorithm in *A* is invoked inside the only main Map function of job *J*. This linear and concurrent aggregated functions are called sub-mappers.
- Sub-Reduce: Like sub-Map, this function is also designed to operate for reducers of all algorithms (i.e., the Reduce function of each algorithm in *A* is implemented separately and invoked in the only main Reduce function of *J*).
- Skew Mitigation: To avoid data skew created from a majority of similar Map output, the comparator function filters intermediate data and schedules them to the Reduce tasks.

### Architecture and Dataset Description

**Fig 1:**
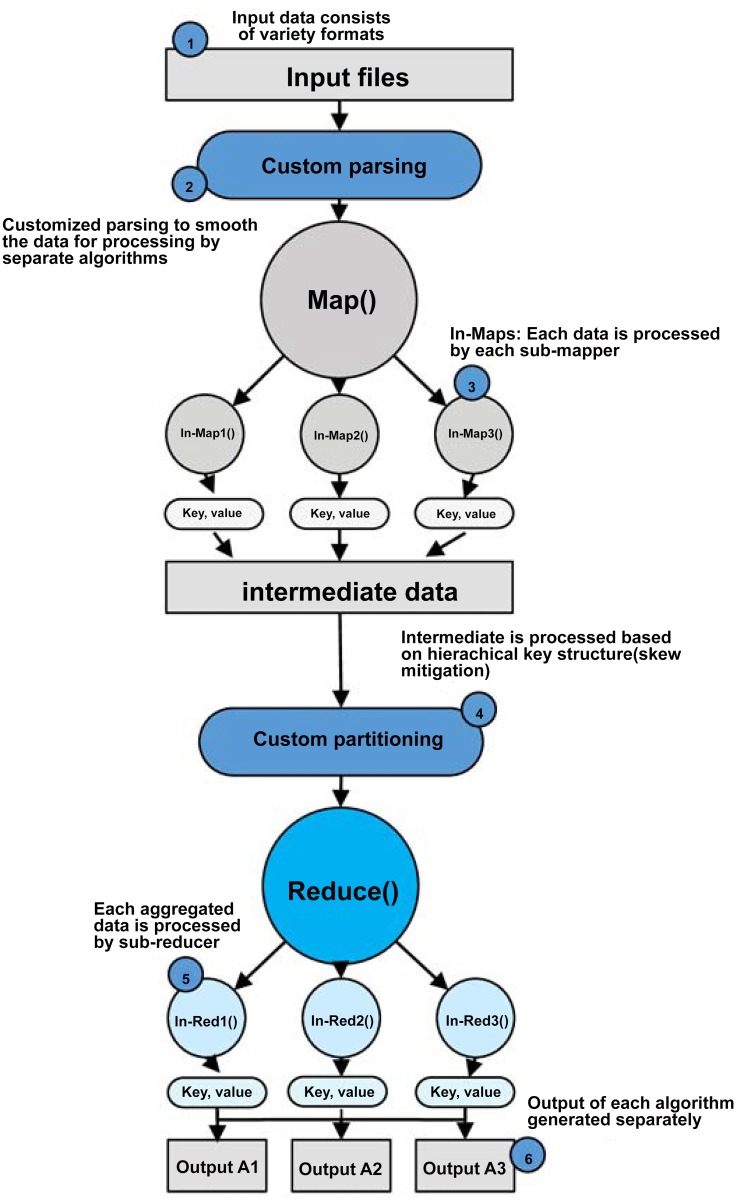
*MRPack* Architecture: The Map and Reduce functions are extended to In-Map and In-Reduce, which function for each algorithm under the umbrella of a single job.

In this section, we initiate the description by In-Map and In-Reduce. Then, we describe the dataset requirements and pre-processing related to *MRPack* followed by the filtering process of intermediate data. In the filtration of intermediate data, we explain the introduction of skew mitigation strategy to avoid large data skew resulting in job delay. We propose a composite key structure to handle multi-algorithm data and skew mitigation. The base architecture of *MRPack* is shown in Fig 1.

In *MRPack*, a MapReduce job is implemented and executed in such a way that it executes multiple related algorithms as a single job. A good example of related algorithms include processing text for natural language processing (NLP) as set, PageRank algorithms as a separate set and machine learning clustering and regression algorithms as a set. All these types of algorithms can process data with slight differences in structure under the same format of data such as textual, or CSV, or tab-separated. The algorithmic dataset requirement and pre-processing, intermediate data management strategy, output generation, and In-Map and In-Reduce algorithm descriptions are discussed in the following sections.

#### Dataset Requirements

To function on *MRPack*, dataset *D* should be formatted according to thebasic requirements of the algorithm. However, the type of data can be different; in unstructured *text*; the type can be a CSV, tab-separated, or space-separated. Similarly, for each algorithm, the dataset can be differentiated based on naming used for files in HDFS repositories. For example, when executing an *InvertedIndex* algorithm, its relevant dataset files in the repository can contain a descriptor to simplify the job of the preprocessor. In chained jobs, the output of one job is used as input of other jobs.

#### Data Preprocessor

As discussed above, the dataset for *MRPack* is filtered being processed by the algorithms. We define a general preprocessor where the input data are preprocessed according to each algorithm requirements. A typical scenario of involving such data processing and filtering is described as below. In a given job *J*, each algorithm in *A* needs to process the subset of data from dataset *D*(*D* = *d*_1_,*d*_2_,*d*_3_,…,*d*_*n*_). Details of the algorithm are discussed in the next section. The preprocessed data are forwarded to the individual algorithm Map function where the Map task output is generated.

#### Intermediate Data Management

Data communication plays a critical role in the performance of MapReduce. Data communication occurs in three stages i.e., data read and write to HDFS, from Map task to Reduce task (intermediate data), and between DataNodes (no-local data existence). These three data movements significantly affect the performance of the cluster. Here we describe our method for handling intermediate data to efficient management and processing.

Intermediate data generated by Map tasks belong to multiple algorithms in a job. These data are differentiable by the *keys* used by each algorithm and are specified in our hierarchical key scheme. In *MRPack*, custom comparator and partitioner functions are implemented to manage intermediate data. In generic MapReduce, these functions are optional; however, they are mandatory in our proposed scheme. The comparator function helps in algorithm specific data filtration and the partitioner function helps with skew mitigation. Based on the keys of each algorithm, we apply a skew mitigation strategy to avoid any delay in the job due to skewed data. We mitigate the data skew by partitioning intermediate data and forwarding it to specific Reducer. In *MRPack*, we use skew mitigation to generate separate output by each Reducer through comparator and partitioner algorithms. The details of comparator and partitioner algorithms are described in the algorithm description section.

#### Output Generation

Efficient management of intermediate data simplifies output generation. To separate the output of each algorithm, we customize “*data writer*” and “*MultipleOutputFiles*” of Hadoop MapReduce, and write all algorithm’s data to a separate file. The separate outputs are necessary to differentiate between the results of each algorithm and reuse the data. These data can be easily reused in iterative algorithms where job chaining is required. The following section describes the algorithms.

### Description of Algorithms

In this section, we describe the *MRPack* detailed algorithms with all its parameters and behaviors. Initially, the preprocessor part of the Map algorithm is described followed by the main *Map* algorithm. Individual sub-Map and sub-Reduce functions specific to each algorithm in the list *A* = *a*_1_,*a*_2_,…,*a*_*n*_ of job *J* are described. At the end of this section, we describe the comparator and partitioner algorithm with composite key approach.

#### Data *Preprocessor* Algorithm

The preliminary part of *MRPack* algorithm is preprocessor. This part filters input data and differentiates between the subsets of data based on naming, data types, and structures. At the start of job *J*, this algorithm reads input from HDFS. This input data *D* is first parsed and processed in the preprocessor as shown in algorithm 1. This algorithm involves the data chunk from *D* which needs to be parsed against the specifications *S* of algorithms in set *A*(*A* = *a*_1_,*a*_2_,*a*_3_,…,*a*_*n*_). If *d*_*i*_ in *D* conforms to *s*_*i*_ in *S*; then, this data chunk is processed by that algorithm. Otherwise, it is skipped to parse the next data chunk. Each data chunk is processed by at least one algorithm and at most by all algorithms. The returned identified sub datasets are processed by individual Mappers of algorithms. This algorithm is invoked by main Mapper of job *J*.

#### Main *Map* Algorithm

The Map function is the basic function of job in *MRPack*. In this function, the data preprocessor is first invoked to filter the data and return it to be processed by specific sub-Map as shown in algorithm 2.

This algorithm shows Mapper of the main job which consists of sub-mappers. A data chunk returned by the preprocessor is processed and *key*−*value* pairs are generated by one or many algorithms as shown by the functions *Mapper*_*A*_ *lg*_1_,…,*Mapper*_*A*_ *lg*_*n*_. These calling functions are individual Mappers of algorithms in *MRPack*. The *sp* in the algorithm indicates specific key configurations of each algorithm. For example, executing a machine learning algorithm such as k-nearest neighbor (*KNN*) requires a text dataset that consists of either comma or tab-separated fields. In this case, the preprocessor algorithm first identifies whether the data chunk is process-able and determines the format with defined algorithm specifications. These data are then processed by individual algorithm (e.g., *kNN*).

**Algorithm 1**: Preprocess Data (*D* = *d*_1_,*d*_2_,*d*_3_,…,*d*_*n*_)

**Data**: *D* = *d*_1_,*d*_2_,*d*_3_,…,*d*_*n*_: Input datasets belonging to algorithms in *A*

*S* = *sp*_1_,*sp*_2_,*sp*_3_,…,*sp*_*n*_: algorithmic specifications;

**Result**: Algorithm specific labeled dataset

1 **for** ∀ *d* ∈ *D* **do**

2       /* check the data chunk */;

3  **if** *D*.*d*_*i*_ = = *sp*_*i*_ **then**

4   **if** *Compliant*(*D*.*d*_*i*_) **then**

5        /* Check data compliance */;

6    *D*.*d*_*i*_.*sp* = *sp*_*i*_;

7      /* Filter the data for compliance */;

8   *D*.*d*_*i*_.*sp* = *Filter*(*D*.*d*_*i*_); *D*.*d*_*i*_ = *D*.*d*_*i*+1_;

9  **else**

10     /* Set to null if the data is not for *sp*_*i*_ */;

11   *D*.*d*_*i*_.*sp* = *null*; *D*.*d*_*i*_ = *D*.*d*_*i*+1_;

12 **return** *D*.*d*_*i*_   /* Return filtered and identified sub dataset */;

**Algorithm 2**: Map(*D* = *d*_1_,*d*_2_,*d*_3_,…,*d*_*n*_)

**Data**: *D* = *d*_1_,*d*_2_,*d*_3_,…,*d*_*n*_: Input data chunks from HDFS

*S* = *sp*_1_,*sp*_2_,*sp*_3_,…,*sp*_*n*_: algorithmic specifications;

**Result**: Intermediate data: key-value pairs

1 **for** ∀ *d* ∈ *D* **do**

2     /* Preprocess the data before Mappers */;

3  *D*.*d*_*i*_.*sp* = *Preprocess*(*D*.*d*_*i*_);

4  **if** *D*.*d*_*i*_.*sp* = = *sp*_1_ **then**

5    /* Invoke individual algorithm Mappers from 1 to *n* */;

6   *Mapper*_*Alg*1(*D*.*d*_*i*_);

7  **if** *D*.*d*_*i*_.*sp* = = *sp*_2_ **then**

8   *Mapper*_*Alg*2(*D*.*d*_*i*_);

9  **if** *D*.*d*_*i*_.*sp* = = *sp*_3_ **then**

10   *Mapper*_*Alg*3(*D*.*d*_*i*_);

11     /* continue till all algorithms are done */;

12  .

13  .

14  .

15  **if** *D*.*d*_*i*_.*sp* = = *sp*_*n*_ **then**

16   *Mapper*_*Alg*_*n*_(*D*.*d*_*i*_);

17  *D*.*d*_*i*_ = *D*.*d*_*i*_+1;

#### Main *Reduce* Algorithm

The Reduce is the primary Reduce function of MapReduce. After pre-processing, Map output generation, and intermediate data shuffling and partitioning; it aggregates the data based on keys using specific Reduce function. Unlike traditional Reduce functions, this algorithm invokes a special function for a set of key-value pairs. The details are shown in algorithm 3.

**Algorithm 3**: Reduce(*key*,*List*[*values*])

**Data**: *Key*−*value* pairs: Intermediate data key-value pairs

*K* = *k*_1_,*k*_2_,*k*_3_,…,*k*_*n*_: Key formats of all algorithms;

**Result**: Final aggregated result in Key-Value pairs

1 **for** ∀ *value* ∈ *List* **do**

2     /* Iterate over the incoming data */;

3  *key* = *value*.*key*; **if** *key* = = *k*_1_ **then**

4    /* Invoke individual algorithm Reducer from 1 to *n*

5   *Reducer*_*Alg*1(*value*);

6  **if** *key* = = *k*_2_ **then**

7   *Reducer*_*Alg*2(*value*);

8  **if** *key* = = *k*_3_ **then**

9   *Reducer*_*Alg*3(*value*);

10     /* continue till all algorithms are done */;

11  .

12  .

13  .

14  **if** *key* = = *k*_*n*_ **then**

15   *Reducer*_*Alg*_*n*_(*value*);

16  *value* = *value*.*next*();

In this algorithm, every time a Reducer receives a *key*−*value* list, it compares the *key* with metadata (algorithm specification) of algorithms and invokes a specific Reduce function. For example, the two algorithms in *A*, *a*_1_ and *a*_2_, have processed input data and generated Map output in the form of *key*−*value* pairs. The main Reduce algorithm receives and verifies the data with keys of all algorithms. If they belong to any algorithm, its Reduce function is invoked to collect the data and process it.

#### *Sub*−*Map* Algorithm

The individual Map functions of each algorithm in *MRPack* function the same as those of generic MapReduce. However, unlike traditional MapReduce, sub-mappers function on part of the whole job (as job has multiple algorithms). The general sketch of a sub-Map is shown in the algorithm 4. Internal instruction set of each algorithm may vary; so we only shown the *ConstructKey* and *GenerateValue* as the computation functions.

**Algorithm 4**: sub-Map(*d* ∈ *D*)

**Data**: *d* ∈ *D* data chunk: Data input preprocessed;

**Result**: Emit data as (Key,Value) pairs

1    /* Construct key from dataset *d* ∈ *D* */;

2 *key* = *Construct*(*d*);

3 /* Process the data according to algorithm specific requirement */;

4 *value* = *GenerateValue*(*d*); /* emit the key and value as pairs */;

5 *emit*(*key*,*value*);

#### *Sub*−*Reduce* Algorithm

Like sub-Map of *MRPack*, sub-Reduce also operates as sub-part of the main Reducer of an *MRPack* job and performs computation only on its own related data. Generally in a Reduce function, the intermediate data are aggregated and collected as shown in algorithm 5. However, in some special cases the data might require some computation such as *kNN* and *K*−*Means* requiring *mean* calculation and neighbors calculations in the Reduce function when implemented as part of this paper.

**Algorithm 5**: sub-Reduce(*key*,*List* < *values* >)

**Data**: *key*−*List* < *values* > pairs: intermediate data;

**Result**: Emit aggregated result as (Key,Value) pairs

1   /* Aggregate values based on same keys *k* ∈ *K* */;

2 **for** ∀ *value* ∈ *List* **do**

3     /* Iterate over the incoming data */;

4  **if** *this*.*key* = = *key* **then**

5   *sum* = *sum* + *value*;

6      /* Process data if needed */;

7  *vals = ComputeVals(sum);* /* Write the results */;

8  *emit*(*key*,*vals*);

#### Comparator and Partitioner Algorithm

This part of the job plays an important role in many aspects such as intermediate data aggregation, sorting, partitioning, and data skew mitigation. The data are sorted by default by comparator based on keys. For partitioning and skew mitigation, we propose algorithm 6, which provides a brief sketch of partitioner. In this algorithm, the *key* is analyzed and then the value is forwarded to the appropriate Reducer. We set a threshold called *λ* to identify the skew mitigation. Suppose, we have three algorithms with keys as *k*_1_,*k*_2_, and *k*_3_. If *k*_1_ > 50%; then, the data of *k*_1_ are diverted to another Reducer. Because the algorithm of *k*_1_ is generating more intermediate data, which in turn can result in data skew and delay of the completion of the job. Selection of threshold value is based on experimentation. This value can be changed according to the cluster environment and specifications.

#### Key Structure and Hierarchy

**Fig 2:**
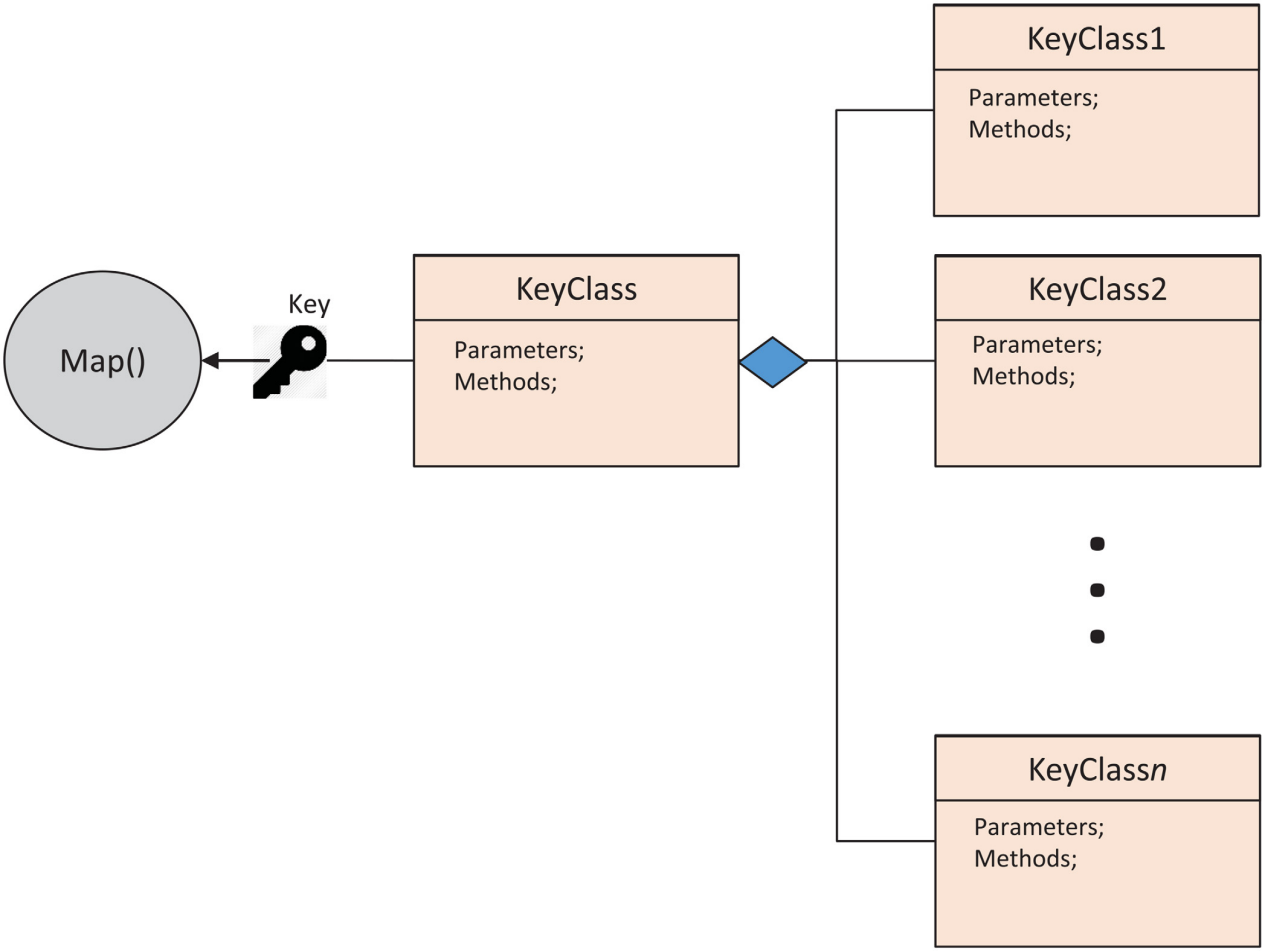
Composite Key Structure: This structure shows keys modeling in *MRPack* where it is used to differentiate the algorithms.

In *MRPack*, maintaining and managing different keys are difficult tasks. Data aggregation, partitioning, and sorting are all based on keys. To efficiently manage keys and overcome the challenge of skewed data, we design a hierarchical and composite key structure. In this scheme, the base class is a general abstract class, and for each algorithm, we extend the base class to use it for special case. We apply polymorphism and composition techniques to handle the keys. The general structure of the keys is shown in Fig 2. The key for each algorithm depends on its specification and requirements.

**Algorithm 6**: Partitioner(*key*,*value*)

**Data**: *key*−*List* < *values* > pairs: intermediate data;

**Result**: Partition data and Reducer allocation

1     /* Analyze *keys* and assign the reducer */;

2 *keyFrequencyList* = *key*; **for** *i* = 0*ton* **do**

3    /* Checks data skew if no skew, directly assign */;

4  **if** *key* = = *k*_*i*_ AND *keyFrequencyList*[*key*] < = *λ* **then**

5   return *i*%(*numOfReducers*);

6  **if** *keyFrequencyList*[*key*] > *λ* **then**

7  /* *λ* is a threshold set for maximum overloaded Reducer */;

8  **for** *j* = 0*ton* **do**

9     /* In case of data skew, check for least used Reducer */;

10   return *leastIndexOf*(*keyFrequencyList*[*k*_*j*_]) % (*numOfReducers*);

### Case Study

**Fig 3:**
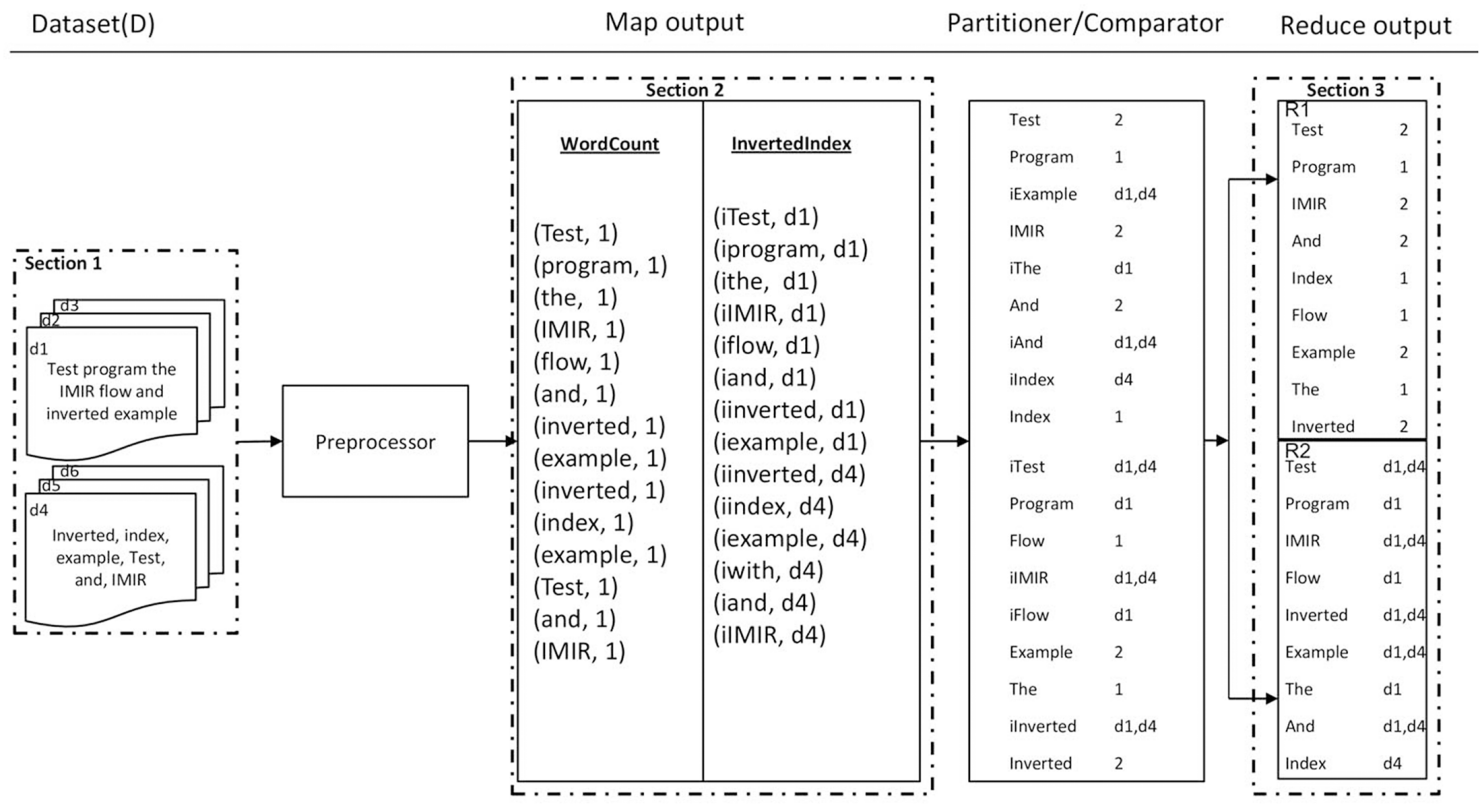
The basic data flow of *MRPack* based on two algorithms, WordCount and InvertedIndex.

In this case study, we explain *MRPack* with two brief and simple algorithms, *InvertedIndex* and *WordCount*. We choose these algorithms because of their simplicity and ease of explanation. The *InvertedIndex* algorithm generates mapping from contents (words) to locations (files), and *WordCount* counts the number of words in a one or all documents. Generally these algorithms can operate on same datasets such as text data, XML, etc. However, for the sake of explanation and overview of the algorithm, we use two types of text data, CSV and tab-separated values. The data structure can vary to any other textual data types such as HTML, JSON, and sensor data. We assume that *WordCount* and *InvertedIndex* both function on all data in order to generate counts and indexes respectively. The sample data are shown in section 1 of Fig 3.

At the start, the *preprocessor* processes the input data and verifies its compliance for the Map step. It breaks the text into words and extracts file names etc. The data are then processed in the Map task of each algorithm and *key*−*value* pairs are generated. Both algorithms use the words as keys, so we need to define a key structure to differentiate between their data. We define a class *Key* with two sub-classes of *WCKey* and *IIKey* for *WordCount* and *InvertedIndex* algorithms respectively. In the Map task, each algorithm generates *key* based on its keys class and the index/count of word as value as shown in section 2 of Fig 3.

In the comparator and partitioner, all intermediate data are scanned and the *key*−*value* pair are determined to belongs to *WordCount*, whether it is forwarded to Reducer1 (R1), or whether it belongs to *InvertedIndex*. Then it is forwarded to Reducer2 (R2 section 3 of Fig 3). In this example, we only define two reducers for the sake of simplicity. The intermediate data will be the same in quantity because both algorithms emit all words as keys. However, in other cases, it might not be necessary to emit each word as *key*. Some algorithms emit a larger number of intermediate data than others, for which we would apply skew mitigation as discussed in the previous sections.

In the last step, the intermediate data are received by the Reducers. Each Reducer can implement each algorithm, so it can process all the data. The partitioner differentiates the data for each algorithm, sending the packets to the appropriate Reducer. Therefore, each Reducer has the output of separate algorithms (see section 3 of Fig 3). Another way of separating the output of each algorithm is the custom implementation of the *datawriter* and *MultipleOutPutFormats*. In *datawriter*, based on the hierarchical key scheme, data can be differentiated.

In this example, the output of each algorithm in the Map step is differentiated by a key as *i* is prefixed to the original word. Similarly, this key is used to partition and sort the data. The comparator can be implemented to work as a sorter, and partial aggregator before sending data to Reducer. This example very briefly explains the whole process. Another implementation contains execution of several clustering algorithms on some dataset such as executing *kNN*,*K*−*Means*,*Bayesian*, or *C*4.5. In this case, set *A* has five algorithms and we need to define five different keys for data differentiation and skew mitigation. In summary, when we increase the complexity of algorithms, number of algorithms, and volume of datasets, the performance of *MRPack* significantly improves and becomes superior to the MapReduce performance. the experimental results aer discussed in section 4.

### Description of Implementation

For implementation, a Hadoop cluster is setup and the algorithm is implemented in JAVA. The cluster setup consists of eight DataNodes with four nodes on physical machines and four on virtual machines (VMs). All VMs have same configuration, while the physical machines have different specifications with respect to memory size and processing power. Each VM has 2GB RAM and minimum of 50GB HDD. Physical machines are used with 4GB RAM and atleast 300GB HDD. Important elements from the implementation are presented as follows:

Algorithm Set: For this implementation, we select kNN, K-Means, WordCount, InvertedIndex, and C4.5. All these algorithms are executed in a single *MRPack* job. We intend to provide a pack of algorithms as part of *MRPack* in this proposed method, which can efficiently be executed in a single MapReduce job.

Keys: For efficient data management, key structure is defined by following the object-oriented programming concept of polymorphism, and individual keys are instances of that class.

Map Algorithm: The main Map algorithm is implemented for the whole job which first executes the pre-process method and then the individual algorithm Mappers are invoked to process the data and generate Key-value pairs.

Partitioner: A custom partitioner partitions the data based on the algorithm and assigns it to a specific reducer.

Reduce: A main Reduce algorithm aggregates the data belonging to a set of keys of an algorithm and writes the output to the HDFS in the form of key-value pairs.

At the initial stage of starting the job, the user has the ability to select an algorithm for chaining and selecting the re-usability of data from the previous job.

### Time and Cost Complexity

This section discusses the time complexity of *MRPack*. For three algorithms *a*_1_,*a*_2_, and *a*_3_ in algorithm set *A* with different costs of complexities, then the complexity of *MRPack* on a single DataNode is the maximum of all three algorithms. The MapReduce algorithm sequentially performs the Map task on a single DataNode. then the complexity of a single Map task becomes the highest in overall job. In the following sections, we explain the computation and communication costs in detail.

#### Communication Cost

Elapsed communication costs consist of data movement from Mappers (*M*) to Reducers (*R*) defined as in-memory data movement. Additional costs in a job are often incurred as result of failing, straggling tasks and data movement between DataNodes. The elapsed communication and additional costs for *n* and *m* number of *M* and *R* respectively is described as.

$$\begin{array}{c} ElapsedCommCost\left(E_{cost}\right)=\sum_{i=0}^{n}M_{i}+\sum_{j=0}^{m}R_{j} \end{array}$$ (1)

The total communication cost consists of elapsed, input-output, and additional cost. Therefore,

$$\begin{array}{c} Totalcomm.Cost\left(T_{com}\right)=M_{size}+E_{cost}+R_{size}+AdditionalCost \end{array}$$ (2)

$$\begin{array}{c} T_{com}=M_{size}+\sum_{i=0}^{n}M_{i}+\sum_{j=0}^{m}R_{j}+R_{szie}+p*\lambda +q*\sigma \end{array}$$ (3)

where *λ* represents the rate of rescheduled straggling tasks in the cluster and *σ* represents the rate of rescheduled failing tasks with p and q representing their count respectively.

#### Computational Cost

In an approximate assumption, an algorithm has complexity *C* executed over dataset *D* with a maximum number of Map tasks *M*_*n*_ and Reduce tasks *R*_*m*_ on a DataNode *D*_*i*_ (maximum number of tasks among all nodes). In *MRPack*, the job is first divided into Map and Reduce steps and then into individual algorithm Mappers and Reducers. Therefore, we estimate the computation cost as the maximum of the cost of Map i.e., *max*(*Map*) and Reduce i.e., *max*(*Reduce*).

$$\begin{array}{c} Computationcost\left(ComplexityC\right)=max\left(Map\right)*C_{m}+max\left(Reduce\right)*C_{R} \end{array}$$ (4)

For example, if *max*(*Map*) and *max*(*Reduce*) are *O*(*n* ^2^) and *O*(*nlogn*) respectively, then

$$\begin{array}{c} Computationcost\left(ComplexityC\right)=O\left(n^{2}\right) \end{array}$$ (5)

The total cost *T*_*cost*_ is finally given by the combination of communication and computation costs as:

$$\begin{array}{c} T_{cost}=T_{com}+C \end{array}$$ (6)

$$\begin{array}{c} T_{cost}=\left\{M_{size}+\sum_{i=0}^{n}M_{i}+\sum_{j=0}^{m}R_{j}+R_{szie}+p*\lambda +q*\sigma \right\}+\left\{M_{n}*C_{m}+R_{m}*C_{R}\right\} \end{array}$$ (7)

This cost remains the same as that of a single MapReduce job, which demonstrates the significant improvement achieved through the use of *MRPack*. The complexity of *MRPack* for the set of algorithms is the same as that of MapReduce for a single algorithm.

## Experimental Evaluation

In this section, the characteristics and performance of *MRPack* are evaluated and compared with those of MapReduce. Real publicly available datasets are used, and artificial datasets are generated for better understanding of the process. Many algorithms are implemented, and the results are explained with weak, and strong points and differences. In all experiments, the time calculation is estimated for I/O time, computation, and intermediate data communication.

### Dataset Description

In the experimental evaluation, three publicly available text datasets and an artificially generated dataset are used. Source code for the experiments is available on [26] and the datasets are described as below.

#### Canadian government dataset

The Canadian government dataset consists of data from the government’s different services including financial and national geographic information [27]. The data are released under the project name “Open Data”. We use the text and CSV format of the data from census of population category for our experimental purpose. This dataset is helpful in extracting word-based statistics even though it is not the focus of this paper.

#### Reuters-21578 and CV1

This is the text dataset most widely used to measure the effectiveness of different systems and applications [28]. It includes datasets related to text categorization, corpus volume 1 (CV1) consisting of stories, and TREC-AP based on press articles. We use text data from [28] and do not perform any type of special information extraction techniques. However, to test the performance of *MRPack*, we process this dataset using the implemented algorithms and compare their performance to that of the generic MapReduce.

#### Machine learning dataset

This is a publicly available dataset consisting of data from various fields including movies, songs, and companies [29]. This dataset is easily usable for machine learning experiments. We use this dataset mainly for processing by the machine learning algorithms such as kNN and KMeans.

#### Artificial dataset

Volume is one of the foundations of the Big Data concept. Currently existing open datasets do not fulfill this requirement because they are very small. An artificial dataset is generated to fulfill the requirement of Big Data’s volume. Therefore, we generate both textual data and machine learning data based on the specification from the publicly available datasets. We perform experiments on varying datasets to accomplish scalability and performance analysis.

### I/O and processing time analysis

In this subsection, we compare *MRPack*’s data loading time and processing time against those of thewidely used generic MapReduce framework. The described dataset and deployment structure are used for this evaluation.

#### Cluster-size based analysis

**Fig 4:**
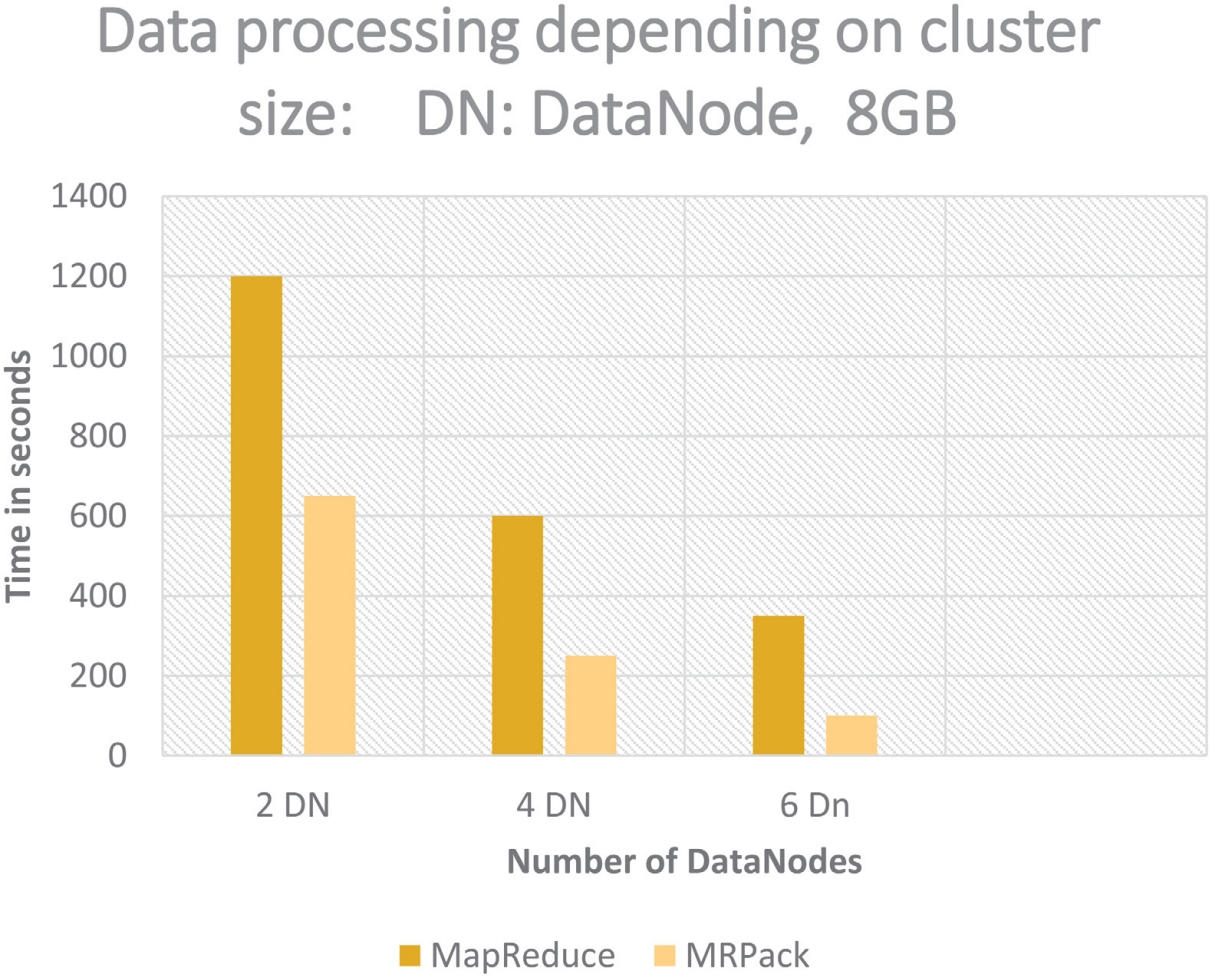
Cluster-based analysis of MRPack performance compared to that of generic MapReduce.

The results of the cluster size experiment are described in Fig 4. The total computation time for each size of cluster is calculated by the NameNode of the Hadoop cluster. The data size is maintained throughout the experiment, and nodes are added and removed after each test. The effect of cluster size is also measured by changing the data volume. With a constant data size at a certain threshold, the performance of the cluster improves by increasing the number of nodes as shown in Fig 4. In this case, when the data size is increased from 2GB to 8GB, the performance of the cluster improves with the addition of extra nodes. Similarly, the data size and cluster size are proportional to each other (i.e., in large datasets, addition of nodes decreases the total time).

#### Data-size analysis

**Fig 5:**
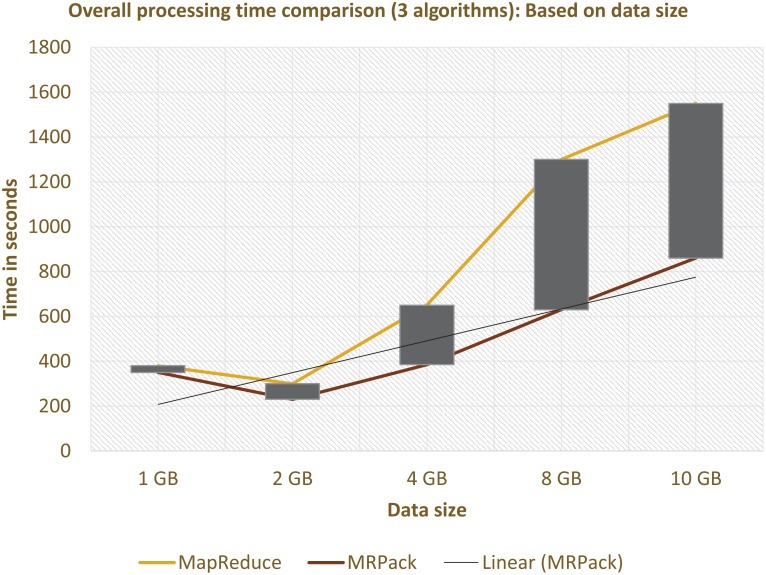
Analysis based on changing data size w.r.t overall job execution time.

In this subsection, we evaluate the performance based on changing data size with constant cluster size. Both *MRPack* and MapReduce are executed on varying datasets and the same cluster consisting of eight nodes. In both cases, after certain threshold of datasets, the performance improved, as shown in Fig 5. However, the generic MapReduce executes a single algorithm in a single pass/job and *MRPack* executes multiple algorithms in a single pass/job. Explicit comparison between both with regard to data size show significant performance improvement for *MRPack*.

#### Algorithm-based analysis

**Fig 6:**
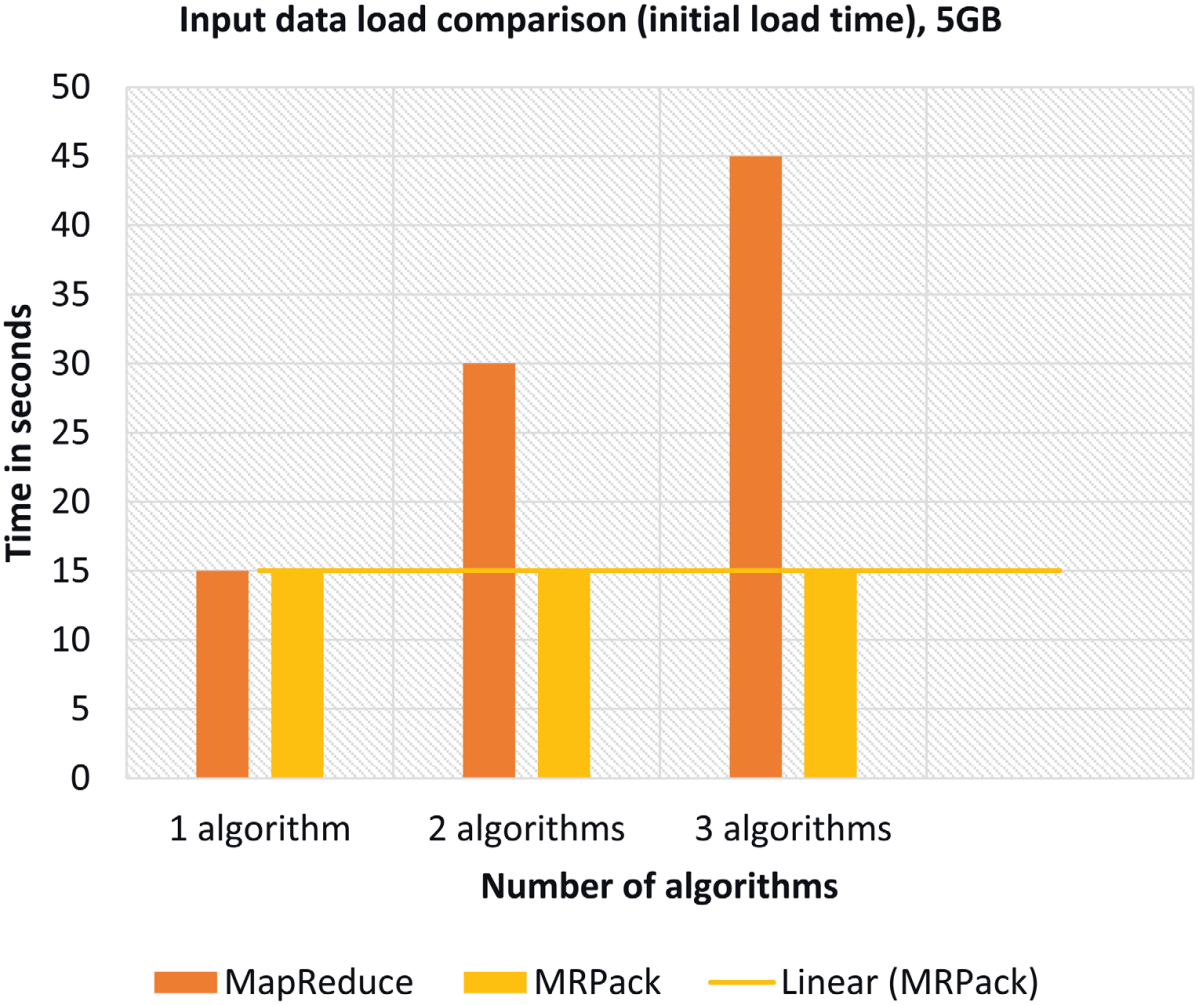
Analysis based on number of algorithms in an MRPack job with respect to time execution time in terms of I/O and network communication.

We test the performance gain and loss when the number of algorithms in *MRPack* varies. In this case, we particularly monitor the I/O communication during job execution with HDFS. In MapReduce, each job requires to retrieve data from HDFS and write it back; hence, it results in long running jobs with heavy I/O operations as shown in Fig 6. However, in *MRPack*, the algorithms are executed as a single job and the I/O operations are performed only once. Hence, the performance is significantly affected and improved compared to that of MapReduce. In MapReduce, when we increase the number of algorithms, the number of jobs to be separately executed also increases. In *MRPack*, increasing the number of algorithms means changing the algorithms only in a single Job. By executing a single job, significant performance improvement is achieved, as shown in Fig 6. However, there are some memory-based limitations to this method, as discussed in the coming sections.

#### Memory stress analysis

Here, we analyze the memory requirements and performance with respect to *MRPack*. Executing multiple algorithms in a single MapReduce job provides significant performance gain in I/O operations, data size, computation, and context switching between jobs. However, it also produces memory stress when the number of algorithms increases a certain limit or the memory of DataNodes is not sufficient. This problem can arise from frequently generated key-value pairs as intermediate data. This limitation can be solved by executing Map and Reduce tasks in parallel, where Reduce tasks process the output of completed Map tasks. The memory stress compared to that of other operations has a low impact on the overall job performance because of its in-memory data movement. I/O, computation, and context switching involve disk read and writes, thus providing a very efficient solution. Scalability of this approach depends on the memory architecture of the cluster. If machines in the cluster hold enough memory contain the intermediate data, then the scalability is the same as that of generic MapReduce. However, in other cases, the limitation of the number of algorithms in the jobs needs to be monitored.

## Conclusion and Future Work

In this paper, we presented *MRPack*, a variant model of Hadoop MapReduce, to support concurrent execution of multiple algorithms in Hadoop. *MRPack* provides an end-to-end MapReduce processing model where computational parallelism is supported with data-parallelism. It provides better performance by processing a single variety dataset with multiple algorithms in a single job through single read and write operations with HDFS. The variety dataset is distributed among DataNodes and processed by at least one algorithm. Intermediate data among various algorithms are differentiated by a novel key-structure. Optimization over the intermediate data processing is performed by incorporating skew mitigation. The results of each algorithm are written back to HDFS in separate files and formats, usable by chained and other jobs. We have bench-marked *MRPack* with MapReduce over varying datasets and algorithms. Our results showed that *MRPack* achieves better performance in data loading, intermediate data management, and data writing. Our results also showed that implementing multiple related algorithms as a single MapReduce job requires less efforts in programming and I/O management. A single implementation of a job is sufficient for a set of related algorithms. Compared to MapReduce, *MRPack* achieves two-fold times performance improvement. This performance ratio varies and depends on the number of algorithms involved in a single *MRPack* implementation.

*MRPack* is an initial step towards performance improvement and computation intensiveness in MapReduce. We intend to continue this research to produce a configurable tool that can be used as software/service with currently available implemented algorithms. We have started working for exploiting Big Data frameworks including MapReduce for real-time data processing using multi-threading. Voluminous data generated every second in a variety of formats requires robust techniques and platforms to receive, link, and manage in a scalable storage system. With real-time data processing, stream data mining and stream data retrieval are the most important aspects that mitigates batch processing systems. We plan to investigate these initiatives and present their outcomes in future research.
