# Correction: MRPack: Multi-Algorithm Execution Using Compute-Intensive Approach in MapReduce

**DOI:** 10.1371/journal.pone.0142761

**Published:** 2015-11-10

**Authors:** The PLOS ONE Staff

Some of the funders listed in the Funding statement did not contribute financially to this study. The publisher apologizes for the error. The correct funding information is as follows: This work was supported by a grant from Kyung Hee University 2013 [KHU-20130438].
